# Quantitative inspiratory–expiratory chest CT findings in COVID-19 survivors at the 6-month follow-up

**DOI:** 10.1038/s41598-022-11237-1

**Published:** 2022-05-05

**Authors:** Xi Jia, Xiaoyu Han, Yukun Cao, Yanqing Fan, Mei Yuan, Yumin Li, Jin Gu, Yuting Zheng, Li Wang, Yali Qu, Heshui Shi

**Affiliations:** 1 Department of Radiology, Union Hospital, Tongji Medical College, Huazhong University of Science and Technology, 1277 Jiefang Avenue, Wuhan, 43002 Hubei People’s Republic of China; 2 Hubei Province Key Laboratory of Molecular Imaging, Wuhan, 430022 People’s Republic of China; 3 Department of Radiology, Wuhan Jinyintan Hospital, No.1 Yintan Road, Dongxihu District, Wuhan, 430022 Hubei People’s Republic of China; 4 Department of Radiology, Wuhan Pingan Healthcare Diagnostic Center, Wuhan, 430022 People’s Republic of China; 5 Department of Function, Wuhan Jinyintan Hospital, No.1 Yintan Road, Dongxihu District, Wuhan, 430022 Hubei People’s Republic of China

**Keywords:** Epidemiology, Influenza virus

## Abstract

We evaluated pulmonary sequelae in COVID-19 survivors by quantitative inspiratory–expiratory chest CT (QCT) and explored abnormal pulmonary diffusion risk factors at the 6-month follow-up. This retrospective study enrolled 205 COVID-19 survivors with baseline CT data and QCT scans at 6-month follow-up. Patients without follow-up pulmonary function tests were excluded. All subjects were divided into group 1 (carbon monoxide diffusion capacity [DL_CO_] < 80% predicted, n = 88) and group 2 (DL_CO_ ≥ 80% predicted, n = 117). Clinical characteristics and lung radiological changes were recorded. Semiquantitative total CT score (0–25) was calculated by adding five lobes scores (0–5) according to the range of lesion involvement (0: no involvement; 1: < 5%; 2: 5–25%; 3: 26–50%; 4: 51–75%; 5: > 75%). Data was analyzed by two-sample t-test, Spearman test, etc. 29% survivors showed air trapping by follow-up QCT. Semiquantitative CT score and QCT parameter of air trapping in group 1 were significantly greater than group 2 (*p* < 0.001). Decreased DL_CO_ was negatively correlated with the follow-up CT score for ground-glass opacity (r = − 0.246, *p* = 0.003), reticulation (r = − 0.206, *p* = 0.002), air trapping (r = − 0.220, *p* = 0.002) and relative lung volume changes (r = − 0.265, *p* = 0.001). COVID-19 survivors with lung diffusion deficits at 6-month follow-up tended to develop air trapping, possibly due to small-airway impairment.

## Introduction

Coronavirus disease 2019 (COVID-19) is a respiratory infectious disease responsible for a global pandemic, and the pathogen has been proven to be severe acute respiratory syndrome coronavirus 2 (SARS-CoV-2)^1^. Globally, as of 27 March 2022, there have been more than 400 million confirmed cases of COVID-19, including more than 6 million deaths, as reported by the World Health Organization (WHO)^2^.

Computed tomography (CT) plays an important role in identifying and investigating suspected COVID-19 patients in the acute phase. Symptomatic and suspected patients should be isolated to control the infection^3^. Some studies^4–6^ have described the clinical characteristics and CT imaging performance in COVID-19 patients. Prevenient publications^5,7–10^ have also demonstrated residual lung function impairment and chest CT abnormalities such as ground-glass opacity (GGO) and fibrosis-like changes in COVID-19 survivors at different time points after discharge. Furthermore, studies by Huang et al.^7^ and Han et al.^8^ of COVID-19 patients 6 months after discharge have reported that greater than 50% of the convalescents had residual chest CT abnormalities. However, studies assessing pulmonary sequelae in COVID-19 survivors by quantitative inspiratory–expiratory chest CT (QCT) are lacking.

Studies^11,12^ reported that air trapping was found in some COVID-19 patients and persisted during the 2-month follow-up. Studies of previous coronavirus infections^13,14^, including severe acute respiratory syndrome (SARS) and Middle East respiratory syndrome (MERS), described the sign of air trapping on CT scanning during convalescence. According to the standard definitions recommended by the Fleischner Society^15^, air trapping is defined as air retention in the distal lung due to pathophysiological obstruction and air trapping on CT is often used to evaluate small-airway diseases (SAD). Although the pulmonary function test (PFT) has been established as the standard method for assessing pulmonary obstructive dysfunction, it appears to be less sensitive to obstructive impairments of small airways^16^. Studies^17–19^ have confirmed that both inspiratory and expiratory CT scans are needed to assess air trapping. In addition, a study^20^ reported that QCT imaging, representing air trapping, is used to assess the functional small-airway disease. However, air trapping evaluated by QCT has rarely been described after COVID-19.

Consequently, this study aimed to assess pulmonary sequelae, especially air trapping, by QCT, leaving aside the other CT findings at the 6-month follow-up. We intended to predict small-airway diseases and explore identifiable risk factors predicting the development of abnormal pulmonary diffusion in COVID-19 survivors at the 6-month follow-up.

We present the following article in accordance with the CONSORT reporting checklist.

## Materials and methods

### Patient population and general information

**Figure 1:**
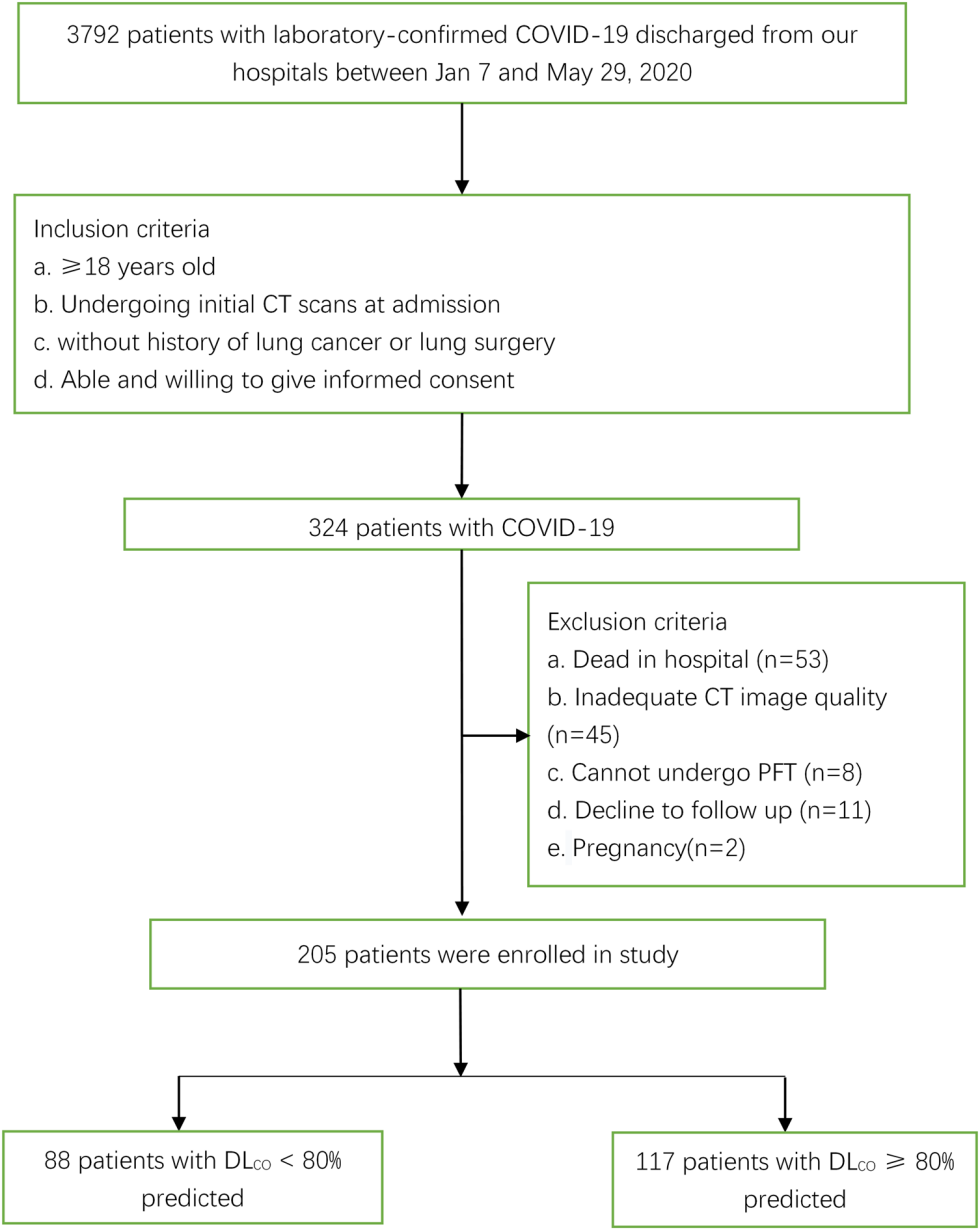
Flow diagram of participant inclusion. *PFT* pulmonary function test, *DL*_*CO*_ carbon monoxide diffusion capacity.

This was a retrospective study. A total of 3792 patients with laboratory-confirmed COVID-19 were discharged from Jinyintan Hospital between January 7 and May 29, 2020. A total of 324 patients were included according to the following inclusion criteria: (1) older than 18 years of age; (2) available initial CT findings at admission; (3) without a history of lung cancer or lung surgery; and (4) able and willing to provide informed consent. A total of 119 patients were excluded because of the following exclusion criteria: (1) death in the hospital (n = 53); (2) inadequate CT image quality (n = 45); (3) inability to undergo PFT at follow-up due to their clinical status (n = 8); (4) declined to follow-up (n = 11); and (5) pregnancy (n = 2) (Fig. 1). The diagnostic criteria for severe pneumonia in adults were in accordance with WHO interim guidlines^21^ and included fever or suspected respiratory tract infection plus one of the following: respiratory rate > 30 breaths/min; SpO_2_ < 90% on room air; or severe respiratory distress. The discharge criteria for all included patients were consistent with the Chinese clinical guidance for COVID-19 pneumonia diagnosis and treatment issued by the National Health Commission^22^. Throat swab specimens from the upper respiratory tract were collected to confirm SARS-CoV-2 by real-time reverse transcription polymerase chain reaction (RT-PCR) using a protocol described previously^7,23^. Clinical data including demographic characteristics, clinical characteristics (onset symptoms, hospital stay duration, self-reported comorbidities, incidence of acute respiratory distress syndrome [ARDS]), peak laboratory findings (the maximum values of parameters reached during the acute phase of the disease), and treatments were collected from electronic medical records by physicians (YKC, XYH, XJ and YTZ, with 7, 5, 3 and 2 years of experience in radiology, respectively). Initial and follow-up CT scans and time from symptom onset to CT scans were also reviewed. We used the Berlin definition of ARDS as a judging reference^24^.

### CT image acquisition parameters

All patients received initial CT scans at admission and completed the QCT at the 6-month follow-up. Only the follow-up QCT was acquired in the expiratory and inspiratory phases, while the initial CT was performed in a single phase. The initial and follow-up CT images were acquired in the supine position using a SOMATOM Definition AS+ scanner or a SOMATOM Perspective scanner (Siemens Healthineers, Forchheim, Germany) with the following parameters: SOMATOM Definition AS+ scanner: pitch, 1.2; collimation, 64 × 0.6 mm; thickens of acquisition, 1.5 mm and mm gap, 1.5 mm, with a reconstruction kernel (B60f); SOMATOM Perspective scanner: pitch, 1/5; collimation, 128 × 0.6 mm/64 × 0.6 mm; thickens of acquisition, 1 mm/5 mm and mm gap, 1 mm/5 mm, with a reconstruction kernel (B80S). The initial CT images of 133/205 (64.9%) patients were reconstructed with a slice thickness of 1 mm and an interval of 1 mm, and those of the remaining 72/205 (35%) patients were reconstructed with a slice thickness of 5 mm and an interval of 5 mm. Due to the decrease in the workload of imaging examination, QCT was performed at the 6-month follow-up, and we deliberately minimized the scan thickness. All follow-up CT images were reconstructed at a 1-mm slice thickness and 1-mm intervals. Noncontrast chest CT scans were performed with acquisition from the thoracic inlet to the diaphragm. Other parameters used for the scanning protocol were as follows: a tube voltage of 120 kV with automatic tube current modulation and a matrix of 512 × 512. The tube current was regulated by an automatic exposure control system (CARE Dose 4D; Siemens Healthineers). All CT images, including the mediastinal window (center, 50; width, 350) and lung window (center, − 600; width, 1200), were obtained using picture archiving and communication systems (Vue PACS, version 11.3.5.8902, Carestream Health, Canada).

### Qualitative CT image evaluation

Three senior cardiothoracic radiologists (HSS, YQF, and JG, with 31, 13 and 10 years of experience in thoracic radiology, respectively) analyzed the CT characteristics without knowing anything about the clinical data, laboratory findings or patient outcomes. Different opinions from the three readers were discussed until a consensus was reached. According to the standard definitions recommended by the Fleischner Society^15^, the predominant pattern on CT scans was categorized as (1) pure GGO, which was defined as increased lung density with no obscuration of the underlying lung marks; (2) fine reticular pattern, which was defined as GGO with reticulation or intralobular networks that were regular, more uniform than crazy-paving pattern, and of the same size; (3) GGO with consolidation, which was defined as increased lung density with obscuration of the underlying lung marks; and (4) mixed pattern, which refers to a combination of consolidation, GGO, and reticular opacities in the presence of architectural distortion and bronchiectasis; (5) air trapping, which was seen on end-expiration CT scans as parenchymal areas with less than a normal increase in attenuation and a lack of volume reduction. To estimate the extent of lung involvement of all these abnormalities, we assigned a semiquantitative CT score to each of five lung lobes. Each lobe was assigned a score from 0 to 5 (0: no involvement; 1: < 5% involvement; 2: 5–25% involvement; 3: 26–50% involvement; 4:  51–75% involvement; 5: > 75% involvement)^25^. The CT scores for the five lung lobes were added to obtain the total CT score, which measured the overall lung involvement, ranging from 0 (no involvement) to 25 (maximum involvement).

### QCT assessment of air trapping

All follow-up CT images were transferred to IntelliSpacePortal software (Version 9.0) for posttreatment. Lung parenchyma was automatically segmented from the chest wall, mediastinum and airways and then analyzed using threshold techniques. The segmentation was adjusted by two physicians (XYH and XJ, with 5 and 3 years of experience in radiology, respectively). Air trapping was quantified using two measures suggested in the current literature.

QCT measures (1) the relative inspiratory to expiratory volume change in voxels with attenuation values from − 860 to − 950 HU (RVC_−860 to −950 HU_). Studies^26,27^ reported that the volume with HU-values below − 950 on inspiratory and expiratory CT was excluded to correct for emphysematous and cystic lesions. RVC_−860 to −950 HU_ is calculated according to the formula expiratory relative lung volume below − 860 HU—inspiratory relative lung volume below − 860 HU, with relative lung volume below − 860 HU defined as the lung volume between − 860 and − 950 divided by the total lung volume over − 950 HU^27^. Increased air trapping causes a higher RVC_−860 to −950 HU_ value.

QCT measures (2) the expiratory to inspiratory ratio of mean lung density (E/I-ratio_MLD_)^28^. Increased air trapping causes a higher E/I-ratio_MLD_.

### Pulmonary function tests

Within 1 week after the 6-month follow-up, PFT was performed and evaluated according to the American Thoracic Society standards on the following items: maximum vital capacity (VC max); forced vital capacity (FVC); forced expiratory volume in one second (FEV1); FEV1/FVC ratio; maximal voluntary ventilation (MVV); DL_CO_; and DL_CO_ divided by the alveolar volume (DL_CO_/VA). All PFTs were measured as a percentage of the predicted value. A measured DL_CO_ < 80% of the predicted value indicated pulmonary diffusion impairment. Patients were divided into group 1 with DL_CO_ < 80% predicted and group 2 with DL_CO_ > 80% predicted.

### Statement of ethical approval

This prospective cohort study was approved by the Ethics Commission of Wuhan Jinyintan Hospital and Wuhan Union Hospital. Written informed consent was obtained from all participants. This trial was registered with the Chinese Clinical Trial Registry, ChiCTR2000038609. The current research was performed in accordance with the Declaration of Helsinki.

### Statistical methods

All of the data were analyzed using SPSS software (SPSS 21.0 for Windows, IBM, Chicago, IL, USA). The data are presented as the median [interquartile range (IQR)] or the mean [standard deviation (SD)] for continuous variables and as counts (percentages) for categorical variables. The chi-square test or Fisher’s exact test was used to compare categorical variables between independent groups. According to the results from the Kolmogorov–Smirnov normality test, the two-sample t-test was performed if the normality test was satisfied. Otherwise, the Mann–Whitney U test was performed if the test results did not indicate normality. Spearman’s rank correlation coefficient was used to evaluate factors associated with DL_CO_. To explore the risk factors associated with abnormal pulmonary diffusion, multiple logistic regression analysis was performed. To prevent data overload, we chose eleven variables that had significant between-group differences as the variables included in the final multiple logistic regression analysis. We included HR, duration of hospital stay, the presence of ARDS, invasive mechanical ventilation and the initial total lesion CT score because there is evidence that these variables are independent predictive factors of fibrotic-like changes in severe COVID-19 survivors^8^. We included d-dimer concentrations because there is emerging evidence of coagulopathy in patients with severe COVID-19^29^. We included the lowest oxygen saturation on room air and the use of glucocorticoids and lactate dehydrogenase (LDH), as these variables were predictors of a 3-month mortality rate of acute exacerbation of idiopathic pulmonary fibrosis^30^. We also included the peak level of leukocyte count and hypersensitive C-reactive protein because of their significant correlation with DL_CO_. A stepwise logistic regression model with a significance level of 0.05 was used in multivariate analysis. The thresholds of each selected variable were based on the medians or the normal medical range, as appropriate. All statistical analyses were two-sided with a significance level of 0.05.

## Results

### Comparison of demographics and initial clinical characteristics

Demographics and clinical characteristics between groups at admission are shown in Table 1. A total of 119 patients were excluded because of the following exclusion criteria: (1) death in the hospital (n = 53); (2) inadequate CT image quality (n = 45); (3) inability to undergo PFT due to their clinical status at follow-up (n = 8); (4) decline to follow-up (n = 11); and (5) pregnancy (n = 2). Finally, 205 patients were enrolled (Fig. 1), including 117 females (57.1%) and 88 males (42.9%), with an age range of 56 ± 12 years. Of the 205 patients enrolled in our study, 80 had severe disease, and 125 had mild disease. All 205 participants enrolled underwent initial CT scans and follow-up CT scans 24 ± 16 days and 200 ± 20 days from symptom onset, respectively. A total of 5 patients reported pulmonary emphysema on admission. The PFT at the 6-month follow-up demonstrated that 88/205 (43%) of the patients had carbon monoxide diffusion capacity (DL_CO_) < 80% of the predicted value (group 1), and the remaining 117/205 (57%) patients had DL_CO_ ≥ 80% of the predicted value (group 2).

**Table 1:** Comparison of demographics and clinical characteristics between groups.

Characteristics	All patients (n = 205)	Group 1 (n = 88)	Group 2 (n = 117)	*p* value
Age, years	56 ± 12	56 ± 12	56 ± 12	0.981
**Sex**				
Female	117/205 (57.1%)	46/88 (52.3%)	71/117(60.7%)	0.256
Male	88/205 (42.9%)	42/88 (47.7%)	46/117 (39.3%)	
Smoking history	36 /195 (18.5%)	15/81 (18.5%)	21/114 (21%)	0.986
History of alcohol consumption	51/195 (26.2%)	18/81 (22.2%)	33/114 (28.9%)	0.292
Fever	169/203 (83.3%)	72/86 (83.7%)	97/117 (82.9)	1.000
Maximum temperature (°C)	38.1 ± 3	38.3 ± 1.0	37.9 ± 3.9	0.462
Cough	153/203 (75.4%)	68/86 (79.2%)	85/117 (72.6%)	0.294
Dyspnea	112/203 (55.2)	55/86 (64%)	57/117 (48.7%)	0.031
HR (bpm)	94 ± 15	98 ± 14	92 ± 16	0.003
Respiratory rate	24 ± 6	25 ± 7	23 ± 4	0.002
SBP (mmHg)	135 ± 19	138 ± 17	132 ± 19	0.045
DBP (mmHg)	84 ± 12	84 ± 10	84 ± 13	0.776
Oxygen saturation on room air (%)	90 ± 11	87 ± 13	92 ± 9	0.001
**Any comorbidities**	114/195 (58.5%)	47/81 (58%)	67/114 (67%)	0.917
Diabetes	32/195 (16.0%)	13/81 (16%)	19/114 (16.7%)	0.909
Hypertension	72 /195 (36.9%)	31/81 (38.3%)	41/114 (36%)	0.742
Bacterial infection	11/201 (5.5%)	6/86 (7%)	5/115 (4.3%)	0.417
Hospital stay duration (days)	25 ± 18	31 ± 21	20 ± 14	< 0.001
ARDS	38/201 (18.9%)	23/86 (26.7%)	15/115 (13%)	0.014
Treatment				
Antiviral agents	163/201 (81.1%)	71/86 (82.6%)	92/115 (80%)	0.647
Antibacterial agents	149/201 (74.1%)	66/86 (76.7%)	83/115 (72.2%)	0.464
Glucocorticoids	71/201 (35.3%)	42/86 (48.8%)	29/115 (25.2%)	0.001
Oxygen therapy	141/201	68/86 (79.1%)	73/115 (63.5%)	0.017
**Mechanical ventilation**				
Noninvasive	33/86 (16.4%)	21/86 (24.4%)	12/115 (10.4%)	0.008
Invasive	6/201 (3%)	5/86 (5.8%)	1/115 (0.9%)	0.042

The data are presented as the means ± SD, medians (interquartile ranges) or n/N (%). *p* values comparing patients with DL_CO_ < 80%(group 1) and patients with DL_CO_ ≥ 80% (group 2) are from χ^2^, Fisher’s exact test, independent-samples T test or Mann–Whitney U test.

*HR* heart rate, *SBP* systolic blood pressure, *DBP* diastolic blood pressure, *ARDS* acute respiratory distress syndrome.

For all clinical presentations, the proportion of patients with dyspnea (group 1: 64% vs. group 2: 48.7%, *p* = 0.031) and the incidence of ARDS (group 1: 26.7% vs. group 2: 13%, *p* = 0.014) in group 1 were obviously greater than those in group 2. Compared with group 2, group 1 had a higher heart rate (HR, 98 ± 14 vs. 92 ± 16, *p* = 0.003), respiratory rate (RR, 25 ± 7 vs. 23 ± 4, *p* = 0.002), and systolic blood pressure (SBP, 138 ± 17 vs. 132 ± 19, *p* = 0.045) and longer hospital stay (31 ± 21 vs. 20 ± 14, *p* < 0.001). Oxygen saturation on room air (%) in group 1 patients at admission was lower than that of group 2 patients (87 ± 13 vs. 92 ± 9, *p* = 0.001). Regarding treatments, participants in group 1 were more inclined to receive glucocorticoids (48.8% vs. 25.2%, *p* = 0.001) and mechanical ventilation, including noninvasive ventilation (24.4% vs. 10.4%, *p* = 0.008) and invasive ventilation (5.8% vs. 0.9%, *p* = 0.042), than those in group 2.

### Comparison of peak laboratory findings

The comparison of laboratory examination data between the two groups is shown in Supplementary Table S1. Leukocyte counts (group 1: 10.7 × 10^9^/L [5.9–14.8] vs. group 2: 6.2 × 10^9^/L [4.8–9.3]), LDH levels (group 1: 428 U/L [298–666] vs. group 2: 304 U/L [229–407]) and D-dimer levels (group 1: 5.36 mg/L [1.4–32.3] vs. group 2: 1.4 mg/L [0.52–4.33]) were obviously higher in group 1 than in group 2 (*p* < 0.001). In group 1, the blood level of hypersensitive C-reactive protein (74.1 mg/L [12.6–153.5]) was greater than that in group 2 (33.8 mg/L [6.1–86.6]) (*p* = 0.004). Hemoglobin levels were significantly decreased in group 1 (109 [96–119]) compared with group 2 (116 [108–127]) (*p* = 0.001). No other significant differences were found between the two groups.

### Comparison of initial and follow-up CT findings and scores

**Figure 2:**
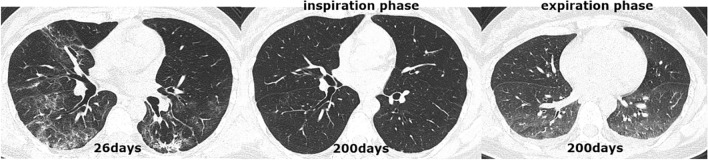
CT scan series in a 40-year-old COVID-19 patient with abnormal DL_CO_ (74.6%) at the 6-month follow-up. (**a**) Transverse CT scan obtained 26 days after the onset of symptoms showed diffuse ground-glass opacities coexisting with consolidations in both lungs. (**b**) Scan obtained during full inspiration on day 200 demonstrated complete resolution of lung abnormalities. (**c**) Scan obtained during expiration at 200 days showed substantial air trapping.

**Figure 3:**
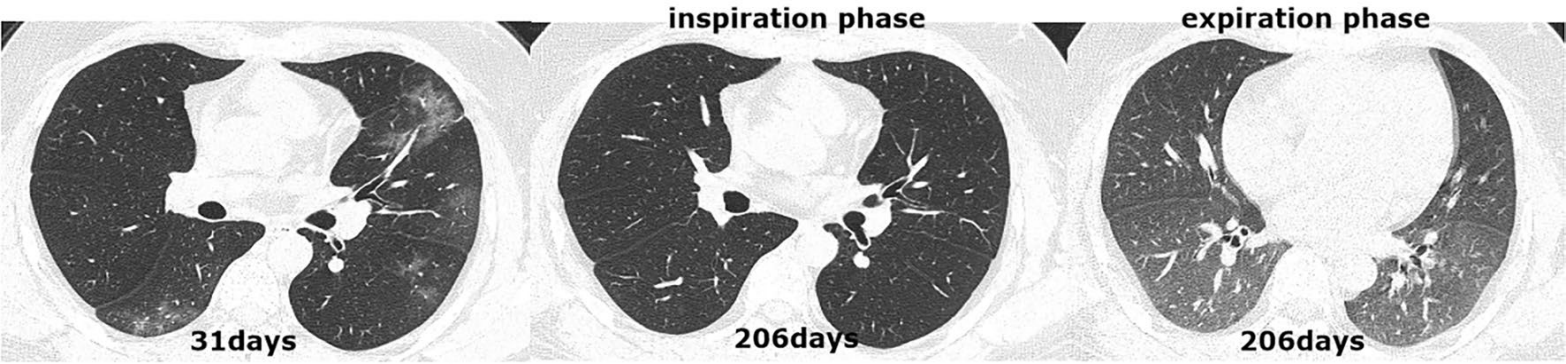
CT scan series in a 65-year-old COVID-19 patient with abnormal DL_CO_ (61.0%) at the six-month follow-up. (**a**) Transverse CT scan obtained 31 days after the onset of symptoms showed multiple consolidations with ground-glass opacities bilaterally. (**b**) Scan obtained during full inspiration on 206 days showed that previous opacifications were markedly dissipated subpleural, irregular linear opacities. (**c**) Scan obtained during expiration at 200 days showed substantial air trapping.

**Figure 4:**
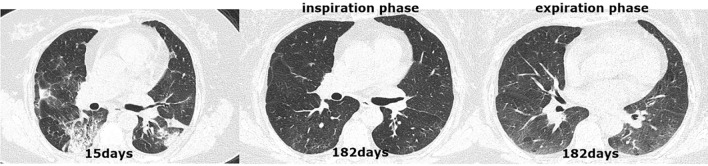
CT scan series in a 37-year-old COVID-19 patient with normal DL_CO_ (107.0%) at the 6-month follow-up. (**a**) Transverse CT scan obtained 15 days after the onset of symptoms showed multifocal ground-glass opacities in the left lung and right lower lobes. (**b**,**c**) Scans obtained during full inspiration and expiration on day 182 showed that previous opacifications were completely absorbed.

All patients received an initial CT scan 24 ± 16 days after symptom onset, and the CT findings and scores are summarized in Table 2. Compared with patients in group 2, those in group 1 had much higher CT scores for total lesions (15 ± 8 vs. 12 ± 7, *p* = 0.004), GGOs (14 ± 8 vs. 11 ± 7, *p* = 0.001) and reticular lesions (5 ± 5 vs. 4 ± 4, *p* = 0.002). As shown in Table 3, all 205 participants completed QCT at the 200 ± 20-day follow-up from symptom onset. The proportion of patients with complete radiological resolution in group 1 was significantly lower than that in group 2 (34% vs. 59%, *p* < 0.001). However, the percentage of patients with complete radiological resolution who had residual air trapping (Fig. 2) was significantly lower than the percentage of patients without air trapping (Fig. 3) (27% vs. 73%). Residual abnormal CT patterns, including those for GGOs, consolidation and reticulation (Fig. 4), were more frequently observed in group 1 than in group 2 (65.9% vs. 41%, *p* = 0.003). Compared with group 2, group 1 had a considerably greater incidence of honeycombing (5% vs. 0.9%, *p* = 0.020). The semiquantitative CT scores of patients in group 1 vs group 2 were obviously higher for total lesions (4 ± 5 vs. 3 ± 4, *p* = 0.007), GGOs (3 ± 4 vs. 1 ± 3, *p* = 0.023), reticular lesions (2 ± 3 vs. 1 ± 2, *p* = 0.015) and air trapping (4 ± 6 vs. 2 ± 3, *p* < 0.001) (Fig. 4). In terms of the quantitative air trapping (QAT) parameters, higher RVC values of the whole lung, right lung and left lung were observed in group 1 than in group 2 (*p* < 0.001). No significant differences were found in E/I-ratio_MLD_ values between the two groups.

**Table 2:** Comparison of initial CT findings and scores between groups.

Characteristics	All patients (n = 205)	Group 1 (n = 88)	Group 2 (n = 117)	*p* value
Time from symptoms onset to CT scan(days)	24 ± 16	26 ± 17	22 ± 15	0.102
**Lung involvement**				
Unilateral	5/205 (2.4%)	3/88 (3.4%)	2/117 (1.7%)	0.653
Bilateral	200/205 (98%)	85/88 (97%)	115/117 (98%)	
**Predominant CT pattern**				
GGO	179/205 (87%)	80/88 (91%)	99/117 (85%)	0.388
Consolidation	15/205 (7.3%)	5/88 (5.7%)	10/117 (8.5%)	
Reticulation	11/205 (5.4%)	3/88 (3.4%)	3/117 (3.4%)	
Presence of nodule or mass	2/205 (1%)	1/88 (1.1%)	1/117 (0.9%)	0.833
Pleural effusion	23/205 (11%)	11/88 (13%)	12/117 (10%)	0.660
Thickening of the adjacent pleura	62/205 (30%)	31/88 (35%)	31/117 (27%)	0.178
Honeycombing	1/205 (0.5%)	1/88 (1.1%)	0/117 (0%)	0.248
Bronchiectasis	11/205 (5.4%)	7/88 (8%)	4/117 (3.4%)	0.212
**CT score**				
Total lesions	13 ± 8	15 ± 8	12 ± 7	0.004
GGO	12 ± 8	14 ± 8	11 ± 7	0.001
Consolidation	4 ± 5	4 ± 4	4 ± 5	0.833
Reticular	4 ± 5	5 ± 5	4 ± 4	0.002

The data are presented as medians (interquartile ranges) or n/N (%). *p* values comparing patients with DLco < 80%(group 1) and patients with DL_CO_ ≥ 80% (group 2) are from χ^2^, Fisher’s exact test, independent-samples T test, or Wilcoxon rank-sum test. *GGO* ground-glass opacities.

**Table 3:** Comparison of follow up CT findings and scores between groups.

Characteristics	All patients (n = 205)	Group 1 (n = 88)	Group 2 (n = 117)	*p* value
Time from symptoms onset to CT scan (days)	200 ± 20	202 ± 23	198 ± 17	0.173
Complete radiological resolution	99/205 (48%)	30/88 (34%)	69/117 (59%)	< 0.001
Air trapping	27/99 (27%)	9/30 (30%)	18/69 (26%)	1.000
**Lung involvement**				0.002
Unilateral	4/205 (2%)	2/88 (2.3%)	2/117 (1.7%)	
Bilateral	102/205 (50%)	56/88 (63.6%)	46/117 (39.3%)	
Normal	99/205 (48%)	30/88 (34.1%)	69/117 (59%)	
**Predominant CT pattern**				0.003
GGO	58/205 (28%)	30/88 (34.1%)	28/117 (23.9%)	
Consolidation	3/205 (1.5%)	1/88 (1.1%)	2/117 (1.7%)	
Reticulation	45/205 (22%)	27/88 (30.7%)	18/117 (15.4%)	
Normal	99/205 (48.3%)	30/88 (34.1%)	69/117 (59%)	
Presence of nodule or mass	15/205 (7.3%)	7/88 (8%)	8/117 (6.8%)	0.761
Thickening of the adjacent pleura	40/205 (19.5%)	22/88 (25%)	18/117 (15.4%)	0.086
Honeycombing	7/205 (3.4%)	6/88 (5%)	1/117 (0.9%)	0.020
Bronchiectasis	28/205 (13.7%)	16/88 (18.2%)	12/117 (10.3%)	0.102
**CT score**				
Total lesions	3 ± 4	4 ± 5	3 ± 4	0.007
GGO	2 ± 4	3 ± 4	1 ± 3	0.023
Consolidation	0 ± 1	0 ± 1	0 ± 0	0.098
Reticular	2 ± 2	2 ± 3	1 ± 2	0.015
Air-trapping	3 ± 5	4 ± 6	2 ± 3	< 0.001
**Quantitative CT parameters**				
**Whole lung**				
RVC	− 0.23 ± 0.10	− 0.20 ± 0.08	− 0.25 ± 0.10	< 0.001
E/I	0.87 ± 0.07	0.87 ± 0.07	0.86 ± 0.06	0.458
**Right lung**				
RVC	− 0.23 ± 0.09	− 0.19 ± 0.08	− 0.25 ± 0.10	< 0.001
E/I	0.87 ± 0.06	0.87 ± 0.07	0.87 ± 0.05	0.414
**Left lung**				
RVC	− 0.24 ± 0.10	− 0.20 ± 0.09	− 0.26 ± 0.10	< 0.001
E/I	0.86 ± 0.07	0.86 ± 0.08	0.85 ± 0.07	0.565

The data are presented as medians (interquartile ranges) or n/N (%). *p* values comparing patients with DL_CO_ < 80%(group 1) and patients with DL_CO_ ≥ 80% (group 2) are from χ^2^, Fisher’s exact test, independent-samples T test, or Wilcoxon rank-sum test.

### Correlation coefficient for DL_CO_

Spearman’s rank correlation analysis (Table 4) revealed significant negative correlations between the impaired DL_CO_ and the initial CT score for GGOs (r = − 0.277, *p* < 0.001) and reticular lesions (r = − 0.199, *p* = 0.004) as well as follow-up CT scores for total lesions (r = − 0.246, *p* < 0.001), GGOs (r = − 0.246, *p* = 0.003), reticular lesions (r = − 0.206, *p* = 0.002) and air trapping (r = − 0.220, *p* = 0.002). Regarding the follow-up QAT measurements, the RVCs of the whole lung (r = − 0.265, *p* = 0.001), right lung (r = − 0.276, *p* = 0.001) and left lung (r = − 0.257, *p* = 0.002) were also negatively correlated with impaired DL_CO_.

**Table 4:** Correlation coefficient for DL_CO_.

Characteristics	Spearman’s correlation coefficient	*p* value
Age, years	0.014	0.838
Sex	0.105	0.136
Heart rate (bpm)	− 0.229	0.004
Oxygen saturation on room air (%)	0.360	< 0.001
Dyspnea	− 0.133	0.059
Duration of hospital stay	− 0.347	< 0.001
ARDS	− 0.250	< 0.001
Noninvasive mechanical ventilation	− 0.111	0.116
Invasive mechanical ventilation	− 0.174	0.014
Glucocorticosteroid use	0.295	< 0.001
Leukocyte count (10^9^/L)	− 0.250	0.001
Hemoglobin	− 0.279	< 0.001
Hypersensitive C-reactive protein (mg/L)	0.247	0.003
Lactate dehydrogenase (U/L)	− 0.332	< 0.001
d-Dimer (mg/L)	0.295	0.001
**CT score of initial CT**		
Total lesions	− 0.206	0.003
CT score of GGO	− 0.277	< 0.001
Reticular	− 0.199	0.004
Complete radiological resolution	0.377	< 0.001
**CT score of follow-up CT**		
Total lesions	− 0.246	< 0.001
GGO	− 0.246	0.003
Reticular	− 0. 206	0.002
Air-trapping	− 0.220	0.002
RVC of whole lung	− 0.265	0.001
RVC of right lung	− 0.276	0.001
RVC of left lung	− 0.257	0.002

All data were analyzed using Spearman correlation. *HR* heart rate, *ARDS* acute respiratory distress syndrome, *GGO* ground-glass opacities.

### Factors associated with abnormal pulmonary diffusion

Multivariate analysis of predictors of abnormal pulmonary diffusion in COVID-19 survivors (Supplementary Table S2) revealed that the minimum value of oxygen saturation on room air < 95% (*p* = 0.037, OR 2.382, 95% CI 1.052–5.397), ARDS (*p* = 0.028, OR 0.229, 95% CI 0.062–0.850) and maximum value of leukocyte count > 10 × 10^9^/L (*p* = 0.023, OR 3.011, 95% CI 1.164–7.784) remained independently correlated with abnormal pulmonary diffusion.

### Follow-up pulmonary function

As shown in Supplementary Table S3, the differences in follow-up pulmonary function indicated that the proportions of patients with VC max%, FVC%, FEV1% and DLCO/V) < 80% predicted in group 1 (DLCO < 80% predicted) were markedly higher than those in group 2 (DLCO ≥ 80% predicted) (p < 0.05). However, no other predicted differences in FEV1/FVC and MVV < 80% were found between the two groups.

## Discussion

As the number of COVID-19 survivors increased, there was growing concern about the pulmonary sequelae of COVID-19 survivors. The present study indicated that 43% of COVID-19 survivors had abnormal lung diffusion capacity (DL_CO_ < 80%) at the 6-month follow-up. The semiquantitative CT scores for air trapping and the quantitative air trapping parameters in patients with DLCO < 80% were obviously higher than in patients with DLCO ≥ 80% at follow-up. Multivariate analysis showed that oxygen saturation on room air < 95%, ARDS and leukocyte count > 10 × 10^9^/L at admission were independent risk factors for abnormal pulmonary diffusion at follow-up, which negatively correlated with the follow-up CT score of GGOs, reticulation and air trapping.

In our study, the frequency of complete radiological resolution at the 6-month follow-up was 48%, higher than 28–38% in other 6-month follow-up studies^8,31^. The discrepancy may be due to the fact that other studies enrolled moderate to severe COVID-19 patients while this study also included mild patients. GGOs and fibrotic-like changes were the top two most frequent findings of follow-up CT abnormalities in this study, which was consistent with other studies^8,31,32^. Furthermore, fibrotic-like changes (like reticulation, honeycombing and bronchiectasis) increased while GGOs reduced on the 6-month follow-up CT when compared with the baseline CT. This variation trend was also consistent with other studies^8,31^ at the 6-month follow-up. It is suggested that fibrotic-like changes maybe the most common CT abnormalities at long-term follow-up. Whether these lesions are reversible needs further research. In addition, several studies^33–35^ in chronic obstructive pulmonary disease (COPD) or chronic airway disease proposed indicators of air trapping by expiratory chest CT scans. Inspiratory and expiratory CT expose patients to additional radiation, but more research is needed to optimize the radiation dose for the quantification of air trapping^36^. However, QCT scans can distinguish air trapping due to emphysema from air trapping due to small airway diseases (SAD)^33^. QCT is superior to expiratory CT imaging alone to define indicators of SAD as predictors of lung function. Not all patients who receive CT scans complete PFT. Patients who recognize SAD can be recommended to undergo PFT for further confirmation.

CT scans demonstrated that significant air trapping existed in approximately one-third of COVID-19 survivors at the 6-month follow-up. Unfortunately, baseline quantitative inspiratory–expiratory chest CT data of all subjects were unavailable. Therefore, patients who had air trapping before being infected with SARS-CoV-2 cannot be excluded. Several studies^37–39^ have suggested that air trapping is mainly caused by emphysema or SAD. In the current study, 5 (2.6%) participants self-reported emphysema at admission. Residual air trapping caused by emphysema cannot be excluded. However, the proportion of patients with emphysema (2.6%) in this cohort was very low, which may have had a relatively small effect on the results. Thus, the residual air trapping in COVID-19 survivors may be mostly due to SAD, which may be related to COVID-19. However, the mechanism of SAD caused by COVID-19 is still unclear. Study^40^ using CT revealed that air trapping was found in part of COVID-19 patients, with rates ranging from 6.1 to 26.3% depending on different length of hospitalization. This semiquantitative assessment of air trapping was different from the QCT assessment in our study, which may be one reason for the difference in results. Autopsy studies^41,42^ of COVID-19 patients reported that SARS-CoV-2 infection can cause acute lung injury, diffuse alveolar damage or virus-induced epithelial changes throughout the airways and alveolar tissue. Small airway diseases were also reported in cured MERS, SARS and ARDS patients at follow-up^13,14,43^. Long-term follow-up studies^13,14^ of SARS survivors showed persistent air trapping on expiratory CT scanning, most likely caused by bronchiolar damage occurring during acute infection and less likely to resolve completely. Likewise, COVID-19 patients at follow-up may also develop air trapping caused by small airway damage. The proportions of residual air trapping in patients with different respiratory virus infections during convalescence are different. Studies^44–46^ of influenza A (H1N1) patients showed that 22–50% of participants had air trapping at different time points from symptom onset, ranging from one month to 3 years. Chang’s study^25^ reported air trapping in 92% (37/40) of SARS survivors at 51.8 ± 20.2 days after symptom onset. Such gaps between studies may be due to the diverse pathogens, follow-up time points and disease severities. CT air trapping can detect SAD, which may cause chronic sequelae of COVID-19, such as interstitial pulmonary fibrosis or emphysema^47^, leading to impaired diffusion capacity. Thus, the assessment of air trapping is important to evaluate the pulmonary prognosis of COVID-19 survivors.

The RVC_−860 to −950 HU_ values in patients with impaired DL_CO_ were significantly higher than those in patients with normal DL_CO_. In addition, RVC_−860 to −950 HU_ values of the whole lung and unilateral lung obtained by QCT had negative correlations with DL_CO_ in the current study. Studies^27,48^ have shown that the RVC_−860 to −950 HU_ value can be used to evaluate the extent of air trapping, excluding emphysematous and cystic lesions. However, the E/I-ratio_MLD_ values were not significantly different between the two groups. This result may be explained by the hypothesis that increased density in some regions at follow-up (for example, due to residual GGOs) could make the E/I ratio a nonsuitable parameter for the quantification of air trapping. This finding may also be due to the E/I-ratio_MLD_ cannot reliably distinguish air trapping from emphysema, which was also reflected by a higher extent of emphysema in people with higher E/I ratios^17^. The results revealed that patients with persisting DL_CO_ deficits were more inclined to develop air trapping. Nevertheless, the correlation between the RVC value and lung diffusion function was relatively weak. Some research^49,50^ reported that anemia, smoking and pulmonary vascular diseases, such as pulmonary hypertension, can also cause a decrease in DL_CO_, which may explain the weak correlation.

Multivariate analysis showed that oxygen saturation on room air < 95% was an independent predictor of abnormal pulmonary diffusion function. One study^51^ demonstrated that the poor oxygenation of COVID-19 patients might be directly related to impaired lung diffusion capacity caused by parenchymal destruction and increased alveolar-capillary distances. Additionally, a higher incidence of ARDS (26.7%) was another independent predictor of lung diffusion dysfunction. Studies^52,53^ have shown that ARDS during acute episodes may lead to the development of chronic lung changes and impaired lung diffusion function. Regarding laboratory tests, the present study found that a leukocyte count > 10 × 10^9^/L was also a risk factor for abnormal pulmonary diffusion function. There is some evidence^54,55^ to suggest that an elevated leukocyte count could be the result of excessive inflammation of lung tissue caused by SARS-CoV-2, subsequently leading to chronic lung disease and abnormal pulmonary diffusion.

The current study revealed that an initial total lesion CT score ≥ 13 was not an independent risk factor for decreased DL_CO_ at follow-up. However, Han et al.^8^ found that a higher CT score (≥ 18) on the initial CT was an independent prognostic factor for the presence of fibrotic-like changes at the 6-month follow-up exam. This disparity in the results could be due to differences in demographics and study criteria. The grouping criteria of Han’s study included fibrotic-like changes in CT scans, which may overestimate the population with true fibrotic lung disease, and only severe COVID-19 patients were included in Han's research. Of the 205 patients enrolled in our study, 80 had severe disease, and 125 had mild disease. Nevertheless, negative correlations between DL_CO_ and the initial CT scores of total lesions, GGOs and reticulation of COVID-19 survivors were found in the current study. Similarly, Hui et al.^56,57^ reported significant negative correlations between abnormal chest radiograph (CXR) scores and DL_CO_ in SARS survivors, reflecting the physiologic effects of lung parenchymal inflammation and fibrosis. Thus, COVID-19 survivors may also have residual lung fibrosis that induces abnormal pulmonary diffusion.

However, there were several limitations in this study. First, the study sample was small, and this study was only a 6-month follow-up study. A larger sample size and longer follow-up would be more ideal to judge the reversibility of lung abnormalities. Second, baseline pulmonary function data and quantitative inspiratory–expiratory chest CT data were unavailable. Patients who had air trapping before being infected with SARS-CoV-2 cannot be excluded. The observed impaired pulmonary function and air trapping cannot be directly attributed to COVID-19. However, the proportion of patients with chronic pulmonary disease in this cohort was very low, which may have had a small effect on the results. Third, 72/205 (35%) patients had a slice thickness of 5 mm in the initial scan, which may prevent subtle findings from being easily noticed. However, all follow-up CT scans were performed with thin slices of 1 mm to evaluate pulmonary abnormalities. Fourth, only patients able and willing to accept follow-up QCT scans were enrolled, which may cause selection biases.

In conclusion, oxygen saturation on room air, ARDS and leukocyte count were identified as independent risk factors for abnormal pulmonary diffusion. Moreover, COVID-19 survivors with persisting lung diffusion deficits at the 6-month follow-up were more likely to develop air trapping, which may be due to small airway impairment. Thus, patients with a low DL_CO_ need QCT scans, which can improve the accuracy of diagnosis, assess disease prognosis and evaluate intervention response early. Longer follow-up studies in a larger population are necessary to understand the reversibility of air trapping and abnormal lung diffusion capacity.

## Supplementary Information

Supplementary Tables.

## Data Availability

All data generated or analyzed during this study are included in this published article.
