# Supplementary material for: Quantitative inspiratory–expiratory chest CT findings in COVID-19 survivors at the 6-month follow-up
Source: Sci Rep. 2022 May 5;12:7402. doi: 10.1038/s41598-022-11237-1 (PMC9070972; doi:10.1038/s41598-022-11237-1)
Supplement: Supplementary file 1 — Supplementary Tables. [file 41598_2022_11237_MOESM1_ESM.docx]

**Quantitative Inspiratory-Expiratory Chest CT Findings in COVID-19 Survivors at the 6-month Follow-up**

**Authors:** Xi Jia^1,2#^, MD, Xiaoyu Han^1,2#^, MD, PhD, Yukun Cao^1,2#^, MD, Yanqing Fan^3^, MD, Mei Yuan^1,2^, MD, Yumin Li^1,2^, MD, PhD, Jin Gu^1,2^, MD, PhD, Yuting Zheng^1,2^, MD, Li Wang^4^, MD, Yali Qu^5*^, MD, Heshui Shi^1,2*^, MD, PhD.

**Addresses:**

1. Department of Radiology, Union Hospital, Tongji Medical College, Huazhong University of Science and Technology, 1277 Jiefang Avenue, Wuhan, Hubei Province 43002, The People’s Republic of China
2. Hubei Province Key Laboratory of Molecular Imaging, Wuhan 430022, The People’s Republic of China
3. Department of Radiology, Wuhan Jinyintan Hospital, No.1 Yintan Road, Dongxihu District, Wuhan City, Hubei Province 430022, The People’s Republic of China
4. Department of Radiology, Wuhan Pingan Healthcare Diagnostic Center, Wuhan 430022, The People’s Republic of China
5. Department of Function, Wuhan Jinyintan Hospital, No.1 Yintan Road, Dongxihu District, Wuhan City, Hubei Province 430022, The People’s Republic of China

**Email:**

Xi Jia: jiaxi981014@163.com

Xiaoyu Han: [xiaoyuhan1123@163.com](mailto:xiaoyuhan1123@163.com)

Yukun Cao: 804423372@qq.com

Yanqing Fan: 1024932023@qq.com

Mei yuan: 549954114@qq.com

Yumin Li: 870802885@qq.com

Ji Gu: [gujin-ll@163.com](mailto:gujin-ll@163.com)

Yuting Zheng: [yuting74029@163.com](mailto:yuting74029@163.com)

Li Wang: wangli19810208@163.com

Yali Qu: 49714972@qq.com

Heshui Shi: heshuishi@hust.edu.cn

**Corresponding Authors:**

Heshui Shi: heshuishi@hust.edu.cn

Yali Qu: 49714972@qq.com

# Xi Jia, Xiaoyu Han and Yukun Cao contributed equally to this work.

* Heshui Shi and Yali Qu contributed equally to this work.

**Supplemental Table S1 Comparison of Peak Laboratory Findings between Groups**

| Characteristics | All patients (n=205) | Group1 (n=88) | Group 2(n=117) | Normal range | *p* value |
| --- | --- | --- | --- | --- | --- |
| Leukocyte count (10^9^/L) | 6.8 (5.3,11.5) | 10.7 (5.9,14.8) | 6.2 (4.8,9.3) | 4-10 | **<0.001** |
| Lymphocyte count (10^9^/L) | 0.8 (0.5,1.2) | 0.78 (0.46,1.05) | 0.78 (0.56,1.31) | 1.1-3.2 | 0.475 |
| Hemoglobin | 113 (100,122) | 109 (96,119) | 116 (108,127) | 110-160 | **0.001** |
| Hypersensitive C-reactive protein (mg/L) | 50 (9.3,114.5) | 74.1 (12.6,153.5) | 33.8 (6.1,86.6) | <25 | **0.004** |
| ESR (mm/h) | 46 (27,70) | 10.4 (7.1,16.3) | 46 (25.2,65) | 0-15 | 0.865 |
| Interleukin-6 (pg/ml) | 9.0 (6.5,13.4) | 46 (29.5,71.6) | 8.6 (6.3,11.9) | 0.1-2.9 | 0.054 |
| Serum amyloid A | 238 (63.9,284) | 216.3 (83.3,284) | 254.5 (59.4,284) | <10 | 0.990 |
| ALT (U/L) | 59 (29,99) | 57 (29.5,100.3) | 60 (29,94) | 8-40 | 0.902 |
| AST (U/L) | 44 (28,67) | 50.5 (31.8,74.5) | 43 (27,63) | 5-40 | 0.140 |
| Lactate dehydrogenase (U/L) | 337 (260,492) | 428 (298,666) | 304 (229,407) | 109-254 | **<0.001** |
| Glucose (mmol/L) | 8.4 (6.5,10.7) | 9.0 (6.3,12.8) | 8.3 (6.5,10.2) | 3.9-6.1 | 0.244 |
| D-dimer (mg/L) | 2.32 (0.7,12.1) | 5.36 (1.4,32.3) | 1.4 (0.52,4.33) | <0.5 | **<0.001** |

The data are presented as medians (interquartile ranges). *p* values comparing patients with DL_CO_＜80%(group 1) and patients with DL_CO_ ≥80% (group 2) are from the Mann-Whitney U test.

ESR, erythrocyte sedimentation rate; ALT, alanine transaminase; AST, aspartate aminotransferase.

**Supplemental Table S2 Multivariable Analysis of Predictors of Abnormal Pulmonary Diffusion in Survivors of Severe COVID-19**

|  | Multivariable Analysis (n=205) | | *p* value | |  |  |
| --- | --- | --- | --- | --- | --- | --- |
|  | Odds Ratio | 95%CI | |  | |  |
| HR >100 bpm | 1.063 | 0.480-2.355 | | 0.880 | |  |
| **Lowest oxygen saturation on room air <95%** | 2.382 | 1.052-5.397 | | **0.037** | |  |
| Duration of hospital stay ≥25 days | 1.023 | 0.998-1.050 | | 0.073 | |  |
| **ARDS** | 0.229 | 0.062-0.850 | | **0.028** | |  |
| Glucocorticoids | 2.361 | 0.959-5.816 | | 0.062 | |  |
| invasive mechanical ventilation | 3.967 | 0.359-2.742 | | 0.580 | |  |
| **Peak Leukocyte count > 10x10^9^/L** | 3.011 | 1.164-7.784 | | **0.023** | |  |
| Hypersensitive C-reactive protein > 25 mg/L | 0.620 | 0.269-1.431 | | 0.263 | |  |
| Lactate dehydrogenase > 254 U/L | 0.884 | 0.331-44.641 | | 0.264 | |  |
| D-dimer > 0.5 mg/L | 2.523 | 0.645-9.878 | | 0.184 | |  |
| Initial total lesions CT score ≥13 | 1.249 | 0.569-2.742 | | 0.580 | | |

HR, heart rate; ARDS, acute respiratory distress syndrome.

**Supplemental Table S3 Comparison of Clinical Characteristics and Pulmonary Function between Groups at the 6-month Follow up**

| Characteristics | All patients (n=205) | | Group 1 (n=88) | | Group 2 (n=117) | | *p* value |
| --- | --- | --- | --- | --- | --- | --- | --- |
| Symptoms |  | |  | |  | |  |
| Dry cough | | 12/205 (5.9%) | | 11/88 (13%) | | 1/117 (0.9%) | **<0.001** |
| Expectoration | | 13/205 (6.3%) | | 12/88 (14%) | | 1/117 (0.9%) | **<0.001** |
| Dyspnea | | 29/205 (14%) | | 25/88 (29%) | | 4/117 (3.4%) | **<0.001** |
| Maximum temperature (℃) | 36.3±0.3 | | 36.3±0.3 | | 36.3±0.3 | | 0.542 |
| Heart rate (bpm) | 81±13 | | 80±13 | | 82±13 | | 0.239 |
| Oxygen saturation on room air (%) | 98±1 | | 98±1 | | 98±1 | | 0.763 |
| Pulmonary Function |  | |  | |  | |  |
| VC max% | 107±15 | | 103±16 | | 109±15 | | 0.003 |
| <80% for predicted | 3/205 (1.5%) | | 3/88 (3.4%) | | 0/117 (0) | | **0.044** |
| FVC% | 108±16 | | 105±17 | | 111±15 | | **0.006** |
| <80% for predicted | 7/205 (3.4%) | | 7/88 (8%) | | 0/117 (0) | | **0.002** |
| FEV1% | 104±17 | | 102±17 | | 106±17 | | 0.131 |
| <80% for predicted | 10/205 (4.9%) | | 8/88 (9.1%) | | 2/117 (1.7%) | | **0.015** |
| FEV_1_/FVC | 94±7 | | 95±8 | | 93±7 | | **0.028** |
| <80% for predicted | 2/205 (1%) | | 0/88 (0) | | 2/117 (1.7%) | | 0.508 |
| MVV | 100±21 | | 94±19 | | 104±21 | | **0.001** |
| <80% for predicted | 22/205 (11%) | | 13/88 (15%) | | 9/117 (7.7%) | | 0.116 |
| DL_CO_ | 85±18 | | 70±8 | | 96±15 | | **<0.001** |
| DL_CO_/VA | 95±16 | | 87±14 | | 101±14 | | **<0.001** |
| <80% for predicted | 35/205 (17%) | | 27/88 (31%) | | 8/117 (6.8%) | | **<0.001** |

The data are presented as the means±SD, medians (interquartile ranges) or n/N (%). *p* values comparing patients with DL_CO_＜80%(group 1) and patients with DL_CO_ ≥80% (group 2) are from χ², Fisher’s exact test, independent vital capacity; DL_CO_, carbon monoxide diffusion capacity; FVC, forced vital capacity; FEV1, forced expiratory volume in 1 s; diffusion capacity of the lung for carbon monoxide; DL_CO_/VA, DL_CO_ divided by the alveolar volume.
